# O1-5 Digital tools for physical activity assessment and brief counselling in Primary Health Care: The Portuguese model

**DOI:** 10.1093/eurpub/ckac094.005

**Published:** 2022-08-29

**Authors:** Catarina Santos Silva, Marlene Nunes Silva, Cristina Albuquerque Godinho, Romeu Mendes, Pedro Teixeira

**Affiliations:** 1 National Program for Physical Activity Promotion - PNPAF, Directorate-General of Health, Lisbon, Portugal; 2 CIPER, Faculdade de Motricidade Humana, Universidade de Lisboa, Lisbon, Portugal; 3 Católica Research Centre for Psychological - Family and Social Wellbeing, Universidade Católica Portuguesa, Lisbon, Portugal; 4 EPIUnit, Instituto de Saúde Pública, Universidade do Porto, Porto, Portugal

**Keywords:** physical activity, sedentary behaviour, brief counselling, primary health care, digital tools

## Abstract

**Issue/problem:**

Implementation of national systems for patients' physical activity (PA) assessment and counselling is a cost-effective strategy recommended in the WHO Global Action Plan for PA 2018-2030. Although Primary Health Care (PHC) professionals are recognized as key agents for PA promotion, challenges remain on how to develop feasible and scalable tools to support them in promoting patients' PA. The Portuguese model could help other countries improve PA assessment and brief counselling in PHC settings. This study aims to describe the tools' development and usage.

**Description of the problem:**

The Portuguese Directorate-General of Health developed two evidence-based digital tools to support PA promotion by healthcare professionals: a) PA brief assessment tool; and b) brief counselling tool. The assessment tool was incorporated within the electronic medical health record software ?SClínico? in September 2017. It includes three questions: 1) how many days per week the patient performs any kind of PA (work, commuting or leisure-time); 2) how much time per day; and 3) how many hours per day the patient spends in sedentary behaviours. The PA brief counselling tool is available through the electronic medical prescription software ?PEM? since December 2017 and consists of five inter-related self-explanatory guides that can be delivered to patients (printed or by email), according to their motivation and PA levels. They facilitate person-centered and autonomy-supportive PA counselling, targeting specific behaviour change mediators, and using validated techniques.

**Results:**

From September 2017 to December 2021, 159,179 patients had their PA assessed (2235 per 100,000 users of the National Health Service) and, from these, 16133 received PA brief counselling guides (177 per 100000 residents in Portugal, ≥ 15 years old), with a six-fold and three-fold increases, respectively, between 2018 and 2019 (previous to the COVID-19 pandemic). Future actions will address cost-effectiveness of this policy.

**Lessons:**

The brief assessment and brief counselling tools were well-accepted and are increasingly being used, with potential for generalized adoption within the Portuguese Health Care System.

**Main messages:**

Portugal has taken a decisive action to promote PA using PHC as a priority setting. PA tools usage is increasing considerably, highlighting the importance of making available easy-to-use PA promotion tools.

